# Immunological Defects in Neonatal Sepsis and Potential Therapeutic Approaches

**DOI:** 10.3389/fped.2017.00014

**Published:** 2017-02-07

**Authors:** Steven L. Raymond, Julie A. Stortz, Juan C. Mira, Shawn D. Larson, James L. Wynn, Lyle L. Moldawer

**Affiliations:** ^1^Department of Surgery, University of Florida College of Medicine, Gainesville, FL, USA; ^2^Department of Pediatrics, University of Florida College of Medicine, Gainesville, FL, USA

**Keywords:** inflammation, innate immunity, shock, infection, host response, genomics, transcriptomics

## Abstract

Despite advances in critical care medicine, neonatal sepsis remains a major cause of morbidity and mortality worldwide, with the greatest risk affecting very low birth weight, preterm neonates. The presentation of neonatal sepsis varies markedly from its presentation in adults, and there is no clear consensus definition of neonatal sepsis. Previous work has demonstrated that when neonates become septic, death can occur rapidly over a matter of hours or days and is generally associated with inflammation, organ injury, and respiratory failure. Studies of the transcriptomic response by neonates to infection and sepsis have led to unique insights into the early proinflammatory and host protective responses to sepsis. Paradoxically, this early inflammatory response in neonates, although lethal, is clearly less robust relative to children and adults. Similarly, the expression of genes involved in host protective immunity, particularly neutrophil function, is also markedly deficient. As a result, neonates have both a diminished inflammatory and protective immune response to infection which may explain their increased risk to infection, and their reduced ability to clear infections. Such studies imply that novel approaches unique to the neonate will be required for the development of both diagnostics and therapeutics in this high at-risk population.

## Introduction

Neonatal sepsis is a major health-care burden worldwide, accounting for approximately 1.4 million neonatal deaths annually (1). Preterm infants are particularly susceptible to sepsis and have a higher risk of long-term complications and mortality than term infants (2). Despite advances in the delivery of neonatal intensive care, improving outcomes as well as diagnostic and prognostic accuracy in neonatal sepsis have been challenging (3).

The diagnosis of neonatal sepsis relies on the subjective interpretation of each case. This stems from a lack of specificity for clinical signs and symptoms (4) and suboptimal predictive ability of routine laboratory tests (5). Even blood cultures, the “gold-standard”, result positive in fewer than 10% of cases of suspected sepsis (6, 7). The subjectivity of the clinicians approach to neonatal sepsis is further demonstrated by the observation that intraregional variation of antibiotic usage in the neonatal intensive care unit can range up to 40-fold (8). Underlying the diagnostic and prognostic challenges for this population is the absence of a consensus definition of neonatal sepsis to align research efforts (2).

The classification of neonatal sepsis is dependent on timing of onset; early-onset neonatal sepsis (EOS) occurs in the first 72 h of life, while late-onset neonatal sepsis (LOS) occurs after the first 72 h and is considered to have been contracted postnatally (9). The primary etiologies of EOS remain group B streptococcal (GBS) infection, despite a reduction in vertical transmission by intrapartum antibiotic prophylaxis, and *Escherichia coli*, which is increasing in very low birth weight (VLBW, <1,500 g birth weight) preterm neonates (3). In contrast, LOS is most commonly caused by coagulase-negative staphylococci and occurs primarily in VLBW neonates (3). The risk factors for EOS are more closely related to vertical maternal transmission (GBS colonization, premature rupture of membranes, maternal urinary tract infection, etc.), while LOS tends to be associated with the shortcomings of long-term critical care hospitalization (invasive procedures, intubation, prolonged indwelling catheters, interruption of natural barriers, etc.), as may be expected in very preterm and extremely preterm neonates (1).

**Figure 1:**
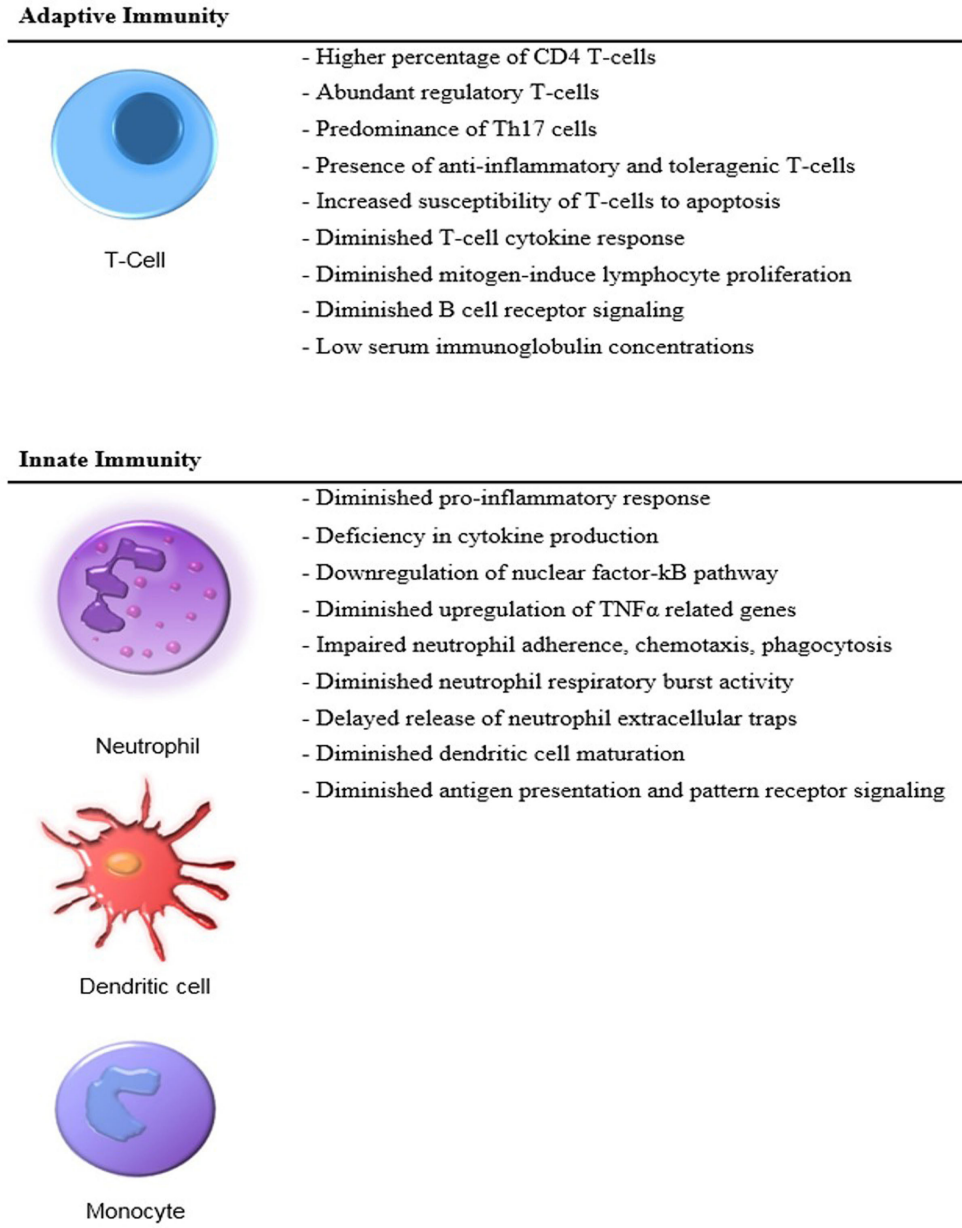
**Distinct features of the neonatal immunity**.

The neonatal adaptive immune system lacks the capacity to support a robust response to infection (10) (Figure 1). The adaptive immune response in neonates differs dramatically from that of children and adults. Neonatal T cells have been categorized as being both anti-inflammatory and toleragenic, a functional phenotype that appears to be programed into the hematopoietic stem cell (HSC) development of neonates (11). Mold and colleagues have shown that different populations of HSCs are active at various stages of development, and neonates possess HSCs whose T cell lineage is biased toward tolerance (12). This premise is also consistent with the findings of Elahi who demonstrated that immature erythroid populations (CD71^+^) found in neonates are immunosuppressive and concluded that these cells increase the risk of infection (13). However, Wynn et al. demonstrated that CD71^+^ erythroid cell depletion, adoptive transfer of CD71^+^ cells, or both (depletion followed by adoptive transfer) had no impact on murine neonatal polymicrobial sepsis survival, and CD71^+^ cells in human neonates were revealed to be enucleated reticulocytes (14).

Because an adaptive immune response favors tolerance and contributes little to host protective immunity, the neonate predominantly relies on an immature innate immunity (15). These deficiencies are linked to the developmental age of the neonate and place the preterm infant at the greatest risk of developing sepsis (16). Despite a significant dependence upon innate immunity, neonates have an underdeveloped innate response to infection including decreased cytokine production and reduced neutrophil and dendritic cell function as compared to adults, which further increases the risk of developing bacterial, fungal, and viral infections (17, 18).

## Transcriptomic Response to Neonatal Sepsis

Since 1990 and the original sequencing of the human genome, the growth of genomic research has greatly expanded our understanding of the underlying response to infection in neonates. In the past decade alone, genome-wide association studies and transcriptomic analyses have become routine and are currently being used for precision medicine initiatives. Genome-wide transcriptomics in particular have become routine discovery tools to better understand tumor and host tissue responses, either by microarray or ribonucleic acid (RNA) sequencing.

With these new tools have come new challenges (19). Bioinformatics have had difficulty keeping up with both the statistical approaches used, as well as the extraction of useable information from these vast amounts of data. Development of biomarkers based on these technologies has been especially challenging due to the initial inability to replicate the findings in confirmatory studies (20). There have been some novel approaches to overcoming the hurdles associated with high-throughput studies conducted in small data sets (21) and the difficulty in replicating the findings. Sweeney et al. have taken a meta-analysis approach using publically available datasets of septic adults and children to extract robust transcriptomic changes that are seen across a large number of individual smaller studies (22, 23).

Investigations into the transcriptomic responses to infection have led to identification of genomic alterations associated with both infection and developmental age (24, 25) and provided new approaches to diagnostics, prognostics, and therapeutics in sepsis (26). In this narrative review, we focus on the current understanding of the neonatal transcriptomic response to sepsis and how this knowledge may be used to improve our investigative and clinical approach to neonatal sepsis.

Among pediatric patients with septic shock, neonates demonstrate significant reduction in the change of gene expression representing key inflammation and immunity signaling pathways compared to infants, toddlers, and children (25). As expected, septic neonates have a significant upregulation, albeit attenuated, of several genes involved in innate immunity compared to uninfected neonates (24, 27, 28). The neonatal innate immune response to sepsis is driven by an increased expression of neutrophils and monocytes (28). By contrast, there is a net suppression of the adaptive immune response as characterized by a decrease in expression levels by T and B cells (28). Genome-wide expression profiles of neonates differ significantly between early- and late-onset sepsis (27), suggesting that the host immune response is determined in part by the postnatal age at the onset of sepsis. Interestingly, genome-wide expression profiles of VLBW neonates with sepsis also differ between Gram-positive and Gram-negative sepsis, suggesting that the neonatal response to sepsis varies depending on the inducing pathogen (24). Similar responses have been seen in adults when exposed *ex vivo* to Gram-negative and Gram-positive pathogens (29).

Studies on the genomic response of whole blood or enriched blood leukocytes from neonates confirm this attenuation of early inflammatory responses. Compared to infants and children, neonates with septic shock downregulate expression of key nuclear factor kappa B pathway-related genes (25). Neonatal mice likewise have an attenuated early inflammatory response to infection compared to adults, including a diminished upregulation of tumor necrosis factor alpha (TNFα)-related genes (30). This raises an interesting paradox. If neonates have a weakened early inflammatory response compared to adults, but early mortality is more common, there can only be a limited number of explanations. The first is that neonates are markedly more sensitive to the inflammatory signals produced early in response to microbial infection. Despite a diminished inflammatory response, that response is much more likely to result in endothelial injury, circulatory failure, organ injury, and death. An alternative explanation is that the attenuation in host inflammatory responses parallels an attenuation of host protective mechanisms. In this case, an early failure to control bacterial growth leads to rapid colonization of organs, cardiovascular failure, and death. Support for this latter hypothesis comes from genomic and functional studies on innate and adaptive immunity in neonates (25, 31). It is likely, however, that both processes are active.

**Figure 2:**
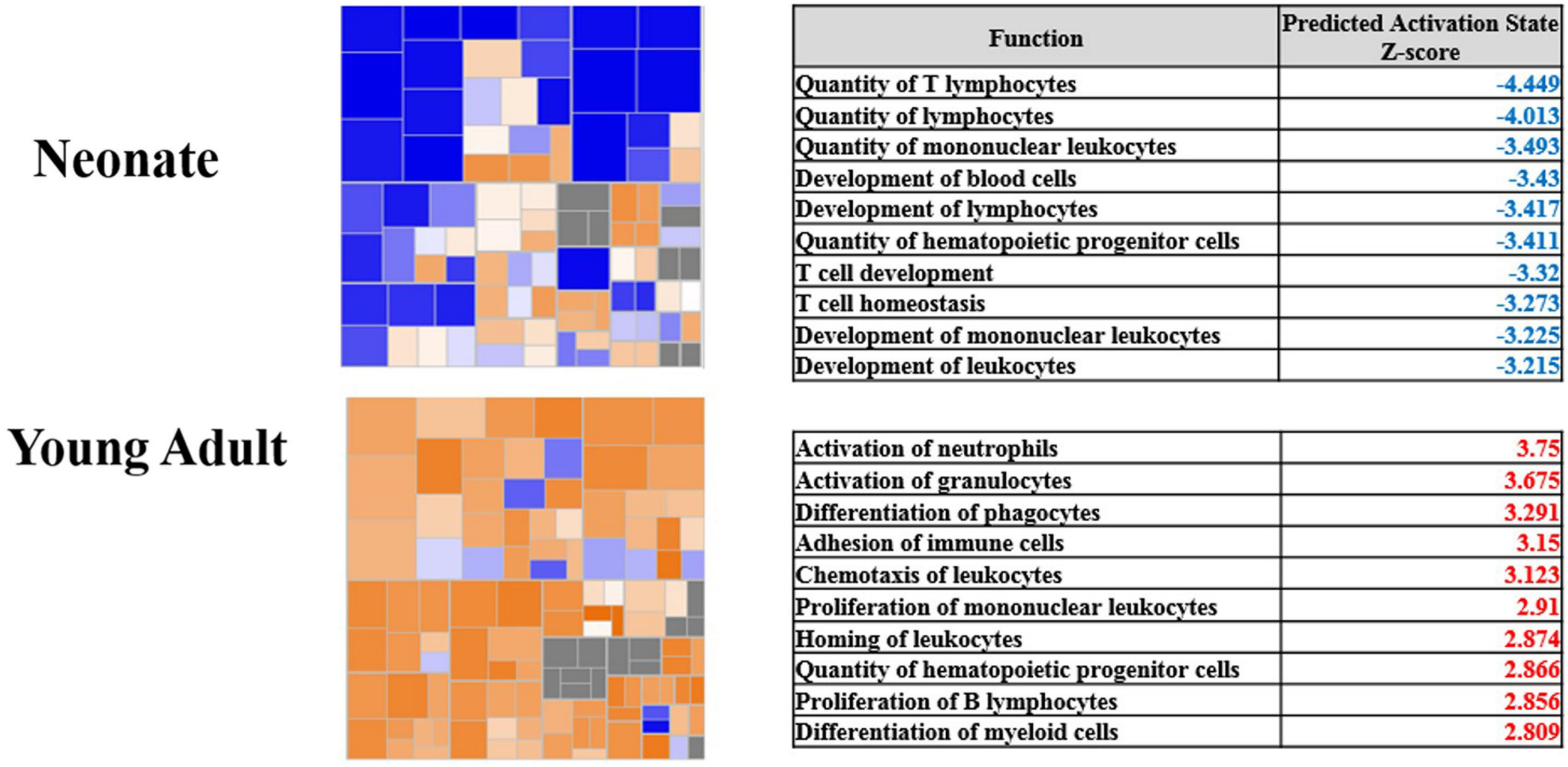
**Pathway analysis of gene expression from blood leukocytes of neonates and young adults subjected to sepsis**. Modified from Gentile et al. (30). Copyright 2014. The American Association of Immunologists, Inc. Heat maps show the gene expression of the functional category hematological systems development and function. Blue represents pathways with an overexpression of genes leading to downregulation of the pathway, whereas orange represents pathways with an overexpression of genes whose activation will lead to upregulation of the pathway. The corresponding tables show the top 10 pathways and their corresponding *z*-score within the functional category. Blue indicates the pathway is significantly downregulated, and red indicates the pathway is significantly upregulated.

The transcriptomic differences seen in human neonates are in agreement with transcriptional changes seen in murine models of polymicrobial sepsis in which neonatal mice exhibit increased mortality compared to young adult mice. These models demonstrate that neonates do not depend on an intact adaptive immune system to provide host protective immunity (15). In response to early sepsis, neonates also have decreased ability to recruit innate immune effector cells to the source of infection, with recruited cells having decreased ability to produce reactive oxygen species compared to adult mice (30). This is best revealed in Figure 2 which examined whole blood leukocyte gene expression from young adult and neonatal mice. Young adult mice had the characteristic increase in the gene expression of proteins involved predominantly in inflammation and innate immunity. Not only were these increases absent in neonatal mice, but there was also a downregulation of genes involved in the development of an adaptive immune response.

Likewise, significant reductions in proinflammatory plasma cytokine concentrations exist among neonatal mice as compared to adult mice including interleukin (IL)-1α, IL-1β, IL-12p40, IL-13, TNFα, granulocyte-macrophage colony-stimulating factor (GM-CSF), macrophage inflammatory protein-1 beta, regulated on activation normal T-cell expressed and secreted, and interferon gamma (32). Analysis 24 h after sepsis reveals neonatal plasma cytokine concentrations are equivalent to young adults except for IL-1β and IL-12p40 which remain diminished. Based on functional pathway analysis, isolated leukocytes from neonatal mice have significant downregulation of pathways 24 h after sepsis corresponding to the development of leukocytes, quantity of T lymphocytes, and proliferation of immune cells (30). In addition to transcriptomic differences in the response to infection, the baseline transcriptome differed significantly between neonatal and adult mice leukocytes by 5,798 probe sets. (30). The representative genes demonstrate functional suppression of the adaptive immunity and downregulation of genes in pathways involved with the activation of the innate immunity.

When evaluating these studies of sepsis in mice, it is important to note key differences between murine and human immunity. Whereas the predominating circulating leukocyte in humans is the neutrophil (50–70%), the lymphocyte is the predominating circulating leukocyte in mice (75–90%) (33). Also, neutrophils are an abundant source of defensins in humans, while mice lack these antimicrobial peptides (34). In addition to differences in innate immunity, humans and mice also exhibit differences in adaptive immunity. These differences include, but are not limited to, expression of Fc receptors, immunoglobulin isotypes and antibody class switching, and T cell receptor signaling and costimulation (35).

Many of these changes in gene expression are recapitulated phenotypically. Neonates exhibit defective neutrophil function compared to adults including adherence, chemotaxis, phagocytosis, neutrophil respiratory burst activity (NRBA), and release of neutrophil extracellular traps (NETs) (36–40). Merry and colleagues demonstrated that neutrophils from healthy neonates have impaired chemotaxis compared to healthy adults and even greater impairment was observed among septic neonates (39). At birth, neutrophil phagocytosis is significantly reduced in both preterm and term neonates as compared to adults; however, this impairment appears to resolve by 3 days after birth (38). This may further explain some of the observed differences in host response to early- and late-onset sepsis, although an additional explanation may be the use of umbilical cord blood (UCB) compared to peripheral blood in this study. UCB may be impacted by administration of antepartum antibiotics as well as the physiological stresses of labor. Healthy preterm neonates have fewer circulating neutrophils compared to term neonates (41) and exhibit a lower percentage of NRBA compared to term neonates and adults (37). In samples from septic patients, both term and preterm neonates demonstrate diminished NRBA. Neonatal neutrophils additionally fail to form NETs following an hour of *ex vivo* lipopolysaccharide (LPS) stimulation, whereas adult neutrophils readily form NETs which bind and kill bacteria extracellularly (40). However, neonatal neutrophils began to form NETs following 2 h of LPS stimulation and nearly reach adult levels by 3 h of stimulation (42).

## Future Directions

Successful modulation of the neonatal immune system to reduce the frequency of sepsis, sepsis mortality, and sepsis survivor morbidity would represent substantial advances in the field. Despite multiple attempts to modulate neonatal immunity in an effort to improve sepsis outcomes in neonates, most therapies have been largely unsuccessful to date (43). This is not particularly surprising given the equivalent failures of immune modulation in adult sepsis, which have prompted clinical trials with rapid molecular assessments of immune status and provision of the appropriate immunomodulatory strategy (anti-inflammatory or immune stimulation) (https://clinicaltrials.gov/ct2/show/NCT02576457). Previous immunomodulatory strategies which have been studied in neonates include granulocyte transfusions, administration of GM-CSF, intravenous immunoglobulin, activated protein C, glutamine, pentoxifylline, anti-endotoxin antibodies, probiotics, and breast milk (43) (Table 1). With the exceptions of breast milk and bovine lactoferrin, these therapies have shown minimal to no reduction in sepsis incidence, and no intervention is associated with an improvement in sepsis-related mortality (43).

**Table 1:** **Summary of strategies aimed at improving the neonatal immune response**.

Strategy	Proposed mechanism	Reference
Granulocyte transfusions	Addition of functional granulocytes	(44)

GM-CSF, G-CSF	Stimulates proliferation, differentiation, and functional activity of myeloid precursors	(45, 46)

Intravenous immunoglobulin	Increase antibody titer and potential antigen–antibody interactions	(47–50)

Activated protein C	Anti-inflammatory and anti-coagulant properties	(51, 52)

Glutamine	Enhance function of immune cells	(53, 54)

Pentoxifylline	Inhibit release of TNFα	(55)

Anti-endotoxin antibodies	Inhibit deleterious effects of endotoxins	(56)

Probiotics	Maintain integrity of the intestinal barrier function	(57)

Breast milk	Provides immunoglobulin A, lactoferrin, oligosaccharides	(58)

Anti-IL17A, anti-IL17A receptor antibodies	Inhibit pathological proinflammatory effects of IL-18	(59)

Toll-like receptor agonists	Augment innate immunity	(15)

Topical emollient	Protect against skin breakdown, prevent pathogens entry	(60)

Lactoferrin	Iron sequestration, disruption of microbial cell membranes	(61)

FFP	Provides humoral immune factors	(62)

*GM-CSF, granulocyte-macrophage colony-stimulating factor; G-CSF, granulocyte colony-stimulating factor; FFP, fresh frozen plasma; IL, interleukin; TNFα, tumor necrosis factor alpha*.

Therapeutics aimed at preventing or treating neonatal sepsis must take into consideration the unique immunological status of the subject. One example of this is the observation that healthy preterm neonates have elevated blood concentrations of IL-18 (63), a proinflammatory member of the IL-1 superfamily. Healthy adults do not manifest high concentrations of circulating IL-18 (64), and their increase in sepsis is markedly lower than the increases seen in neonates (65). Targeting the IL-18/IL-17A axis through the use of neutralizing IL-17 receptor antibodies represents a novel approach to treating neonatal sepsis (59). While IL-18 is known to exert proinflammatory effects such as increased neutrophil phagocytosis and production of reactive oxygen species, paradoxically, IL-18-null (IL-18^−/−^) neonatal mice demonstrated markedly improved survival and reduced bacteremia in comparison to wild-type neonatal mice when challenged with polymicrobial sepsis (59). Unlike human preterm neonates, healthy neonatal mice do not exhibit increased circulating IL-18. When IL-18 was given to septic mice to mimic the human condition, mortality was dramatically increased compared to septic mice alone and was associated with increased bacteremia, intestinal injury, and an increased systemic inflammatory response predominated by IL-17A. The deleterious effects of IL-18 on neonatal sepsis survival were dependent upon IL-1R1 signaling and IL-17A production by gamma delta cells in the intestine and lung (59). Compared to wild-type or isotype control treated mice, transgenic mice lacking IL-17A or wild-type mice that received antibody-mediated receptor blockade of IL-17A through IL-17A receptor or cytokine neutralizing antibodies exhibited markedly reduced mortality to sepsis (59). Currently, two antibodies directed against IL-17A (secukinumab and ixekizumab) have obtained FDA approval for use in other inflammatory diseases, and an additional antibody against IL-17A receptor (brodalumab) is currently awaiting final FDA approval. These therapies may prove beneficial in septic neonates.

The prevention rather than treatment of sepsis is an alternative approach and would be expected to have a significant benefit over the current reactive paradigm. Defined exposure to non-infectious components of bacteria that are recognized by the innate immune system is one approach being considered as a means to positively modify neonatal immune responses. Of the adjuvant therapies currently being studied, toll-like receptor (TLR) agonists appear most promising (66). TLRs play a vital role in early recognition of microbial invasion and activation of the innate immunity (67). TLRs are membrane-spanning receptors present on the cell surface and within intracellular vesicles of leukocytes and other non-immune cell populations such as endothelial cells and fibroblasts (68). Stimulation of these receptors occurs through the binding of ligands, specifically, pathogen-associated molecular patterns (PAMPs) (69). PAMPs may include components of either the bacterial or fungal cell wall or membrane such as LPS, peptidoglycan, or flagellin, or intracellular components such as single- or double-stranded RNA or deoxyribonucleic acid. Activation of these receptors induces downstream molecular signaling events which ultimately triggers the production of inflammatory cytokines, type I interferons, chemokines, and antimicrobial peptides (70).

Toll-like receptor agonists, in particular those affecting TLR 4 and TLR 7/8, have been shown to augment innate immunity, magnify but abbreviate the early systemic inflammatory response, reduce bacteremia, and increase survival to polymicrobial sepsis in neonatal murine models (15). Burl and colleagues also demonstrated that *in vitro* stimulation of newborn cord blood with TLR agonists led to significant production of TNFα, IL-6, IL-1β, and IL-10 (71). Interestingly, several first-generation inactivated and attenuated vaccines including rabies, typhoid, and Bacillus Calmette–Guérin (BCG) possess inherent TLR activity (72). Hence, the inclusion of adjuvants in vaccines has provided non-specific heterologous benefits and enhanced immune responses in traditionally poor-responding populations such as neonates. When BCG was administered at birth to newborns in sub-Saharan Africa, there was a 41% reduction in all-cause mortality at 12 months among VLBW neonates, which was attributed to fewer cases of neonatal sepsis, respiratory infections, and fever (73).

## Conclusion

Neonatal sepsis is prominent cause of morbidity and mortality. A clear understanding of the neonatal response to sepsis represents a critical knowledge gap that greatly limits the opportunity to discover novel diagnostics and therapies to treat and potentially prevent this devastating disease. Evaluation of the transcriptomic response of blood leukocyte populations offers both a global view of the neonatal response to infection and sepsis, and the potential for identification of novel therapeutic opportunities, diagnostic tests, and prognostic markers. Accurate biomarkers would facilitate both targeted treatment, thus avoiding the overuse of empiric antibiotics in non-septic neonates, and an enrichment strategy that facilitates better selection of study participants for future clinical trials.

## Author Contributions

All the authors contributed extensively to the work presented in this paper. SR, JS, and JM wrote the manuscript. SL, JW, and LM gave conceptual advice and edited the manuscript.

## Conflict of Interest Statement

The authors declare that the research was conducted in the absence of any commercial or financial relationships that could be construed as a potential conflict of interest.
